# Infectious keratitis in Western New York: a 10-year review of patient demographics, clinical management, and treatment failure

**DOI:** 10.3389/fopht.2024.1469966

**Published:** 2024-12-11

**Authors:** Caroline Maretz, Jason Atlas, Shalini Shah, Michael B. Sohn, Rachel A. F. Wozniak

**Affiliations:** ^1^ Department of Ophthalmology, University of Rochester School of Medicine and Dentistry, Rochester, NY, United States; ^2^ Department of Biostatistics and Computational Biology, University of Rochester School of Medicine and Dentistry, Rochester, NY, United States

**Keywords:** infectious keratitis, epidemiology, patient outcomes, cornea, social determinants of health

## Abstract

**Background:**

Infectious keratitis (IK) is a blinding disease and an important cause of ocular morbidity. Understanding regional trends in IK are important to understand the epidemiology and clinical outcomes of this disease.

**Methods:**

In this 10-year retrospective review, patient characteristics including sociodemographic factors, medical history, and ocular history were collected as well as the clinical course and outcomes. This study particularly focused on these characteristics as it relates to treatment failure in IK, as defined as requiring more than 2 weeks to heal or surgical intervention, likelihood of having microbiology cultures collected, surgical intervention, and presenting disease severity.

**Results:**

935 cases of IK were identified at the University of Rochester. Age (p=0.004), history of prior corneal transplant (p=0.009), severe vision loss on presentation (p<0.001), large ulcer size (p=0.001), and fungal (p=0.001) or protozoan (p=0.009) infections were all significantly associated with treatment failure. Both ulcer size (p<0.001) and severity of vision loss (p<0.001) were associated with a higher likelihood of having microbiology cultures as well as surgical intervention. Patients’ whose home address was greater than 60 miles from the University were also more likely to present with a more severe ulcer (p<0.001) and undergo a surgical intervention (p=0.05). In studying the impact of race and ethnicity, Black patients were less likely to receive corneal cultures compared to White patients (p=0.02).

**Conclusions:**

This study defined the patient characteristics and clinical course of patients with IK over 10 years at the University of Rochester providing insight into regional trends of the patient population as well as clinical outcomes.

## Introduction

Infectious Keratitis (IK) is a serious, sight-threatening disease of the cornea caused by bacteria, viruses, fungi, or protozoa, and is the fifth leading cause of overall blindness worldwide (1–3). In the United States and other developed nations, studies of IK epidemiology estimate 2.5-27.6 per 100 000 individuals per year (4–6). However, in developing countries, the incidence has been reported as high as 113-799/100 000 (2, 4, 7, 8). Despite these substantial described numbers, it is widely agreed that the true incidence of IK is likely much higher due to limitations in reporting.

The most significant predisposing risk factors for IK include trauma, contact lens wear, ocular surface disease, prior ocular surgery, and immunosuppression, all of which impact the integrity of the ocular surface through perturbations of corneal architecture, innervation, and tear film stability or composition (9, 10). In Asia and other developing nations, ocular trauma remains the most significant reported risk factor for IK, particularly in the setting of agricultural occupations (4, 11–14). In contrast, in the developed world, contact lens wear is the most significant risk factor, particularly among patients who utilize extended or overnight-wear lenses (15). In fact, it has been estimated that contact lens wear accounts for approximately 35-65% of all corneal infections in major US hospitals (16–18). With approximately 45 million contact lens wearers in the US coupled with 99% of surveyed contact lens wearers reporting at least one contact lens hygiene behavior associated with an increased risk of infection (19), it is likely that cases of contact-lens associated IK will continue to rise.

In addition to contact lens wear, ocular surface disease (OSD), such as dry eye, blepharitis and neurotrophic keratitis, has also been associated with developing IK (20), as has prior ocular surgery, even well outside of the immediate post-operative period (21, 22). In the latter, the risk appears the highest in cases of corneal transplantation, likely due to chronic ocular surface perturbations and long-term steroid use; recent studies have suggested that up to 5.9% of patients develop IK after penetrating keratoplasty and 1.3% of patients after endothelial keratoplasty (21, 22). Lastly, immunosuppression due to systemic disease states or medication use may increase the risk of IK. For example, topical steroid use has been identified in up to 13-24% of patients with IK (23, 24). Systemically, patients with HIV have a 9-fold greater risk for developing IK compared to HIV-negative patients (18), and several studies have found that diabetes is associated with greater rates of bacterial and fungal keratitis as well as higher recurrence risk with herpes simplex keratitis (14, 25, 26).

In contrast to ocular risk factors as described above, the relationship between IK and sociodemographic variables and social determinants of health such as age, sex, race, ethnicity, insurance status, primary language, housing, education, employment, and food security are now emerging as important considerations in understanding disease risk and clinical outcomes. While initial epidemiology studies have indicated little sex predilection for developing IK, older age has been repeatedly linked to higher incidence of disease (23, 27–29). Patients’ home address may also significantly impact IK severity at presentation with regards to environmental factors such as pollution as well as living in neighborhoods with low socioeconomic status and limited transportation or access to health services (30–32). Importantly, race has been linked to differences in the number of diagnostic tests offered as well as number of clinical visits in IK suggesting that implicit and explicit bias may also play a role in disease outcomes (33).

As populations and environments vary widely based on geography and community resources, it is important to study regional trends in IK with a wide lens, reporting on both well known risk factors as well as consideration for broader factors such as socioeconomic variables. As such, this is the first study to explore a comprehensive set of IK risk factors and clinical outcomes in Western New York. In this 10-year retrospective study, risk factors for disease as well as subsequent treatment failure in IK are reported with respect to traditional variables such as contact lens wear, trauma, history of ocular surgery, and immunosuppression, as well as epidemiologic variables such as race, ethnicity, primary language, insurance status and geographic distance from medical care to provide an inclusive risk profile of IK in this region.

## Methods

In this retrospective study, electronic health records were obtained for all patients with infectious keratitis between April 2012 and May 2022 at the University of Rochester Medical Center (URMC). URMC’s institutional review board for human subject research granted approval for the study.

**Figure 1:**
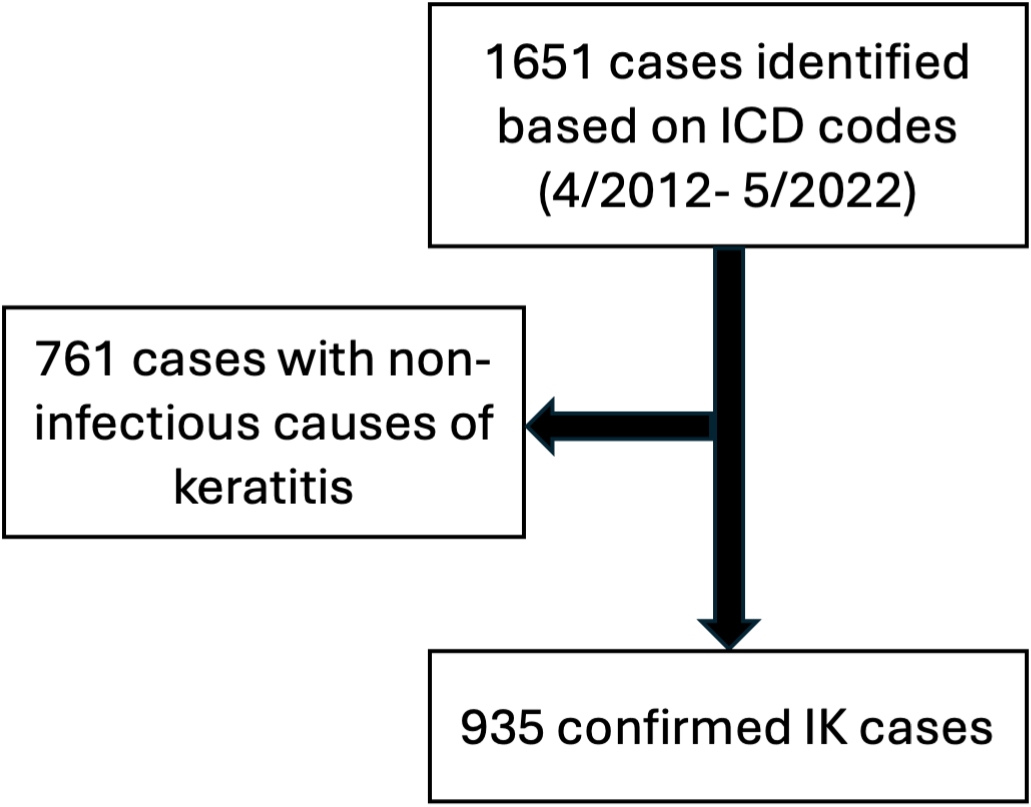
Schematic of included patients in this study.

To identify eligible patients, all clinical encounters that included a diagnosis of “keratitis”, “corneal ulcer”, “eye infection” and related terms, were identified via International Classification of Disease (ICD) codes. The ICD coding system was updated from ICD 9 to ICD 10 on October 1^st^ 2015, and so 20 ICD-9 codes and the equivalent 66 ICD-10 were used in total (**Supplementary Table S1**). Patients were included only if the etiology of keratitis due to an infectious organism (bacteria, virus, fungus, acanthamoeba); however, patients were not required to have culture-confirmed IK and a clinical diagnosis of infectious keratitis was sufficient for inclusion in the study. This resulted in a total of 935 patients who met inclusion criteria (**Figure 1**).

Data collection included demographic, medical history, ocular history as well as the entire clinical course until completion of treatment (including cessation of antibiotics). Demographic data included patient age, sex, race, ethnicity, primary language, zip code and health insurance coverage (private carriers, Medicare, Medicaid and self-pay). Medical history included hypertension, diabetes status as well as degree of diabetes control based on HbA1c levels <7%, smoking status (>5 pack-year history), and immunosuppressive medications such as systemic steroids, methotrexate, azathioprine, biologics (ex. Adalimumab) and current chemotherapy regimens. Patients’ ocular history included contact lens use, prior surgeries, ocular trauma or other diagnoses, and topical medications at the time of presentation. Visual acuity at baseline (prior to IK, if known), at the time of presentation of IK, and at the visit at which IK had fully resolved, slit lamp exam findings (including ulcer size and anterior chamber reaction) at presentation, prescribed medications and surgical interventions, length of treatment (in days), and whether the treatment resulted in a successful outcome, defined as improvement clinically (complete epithelialization of the corneal ulcer and resolution of active stromal infiltrate), within two weeks of the initial diagnosis and no surgical intervention, were documented. Conversely, treatment failure was noted if patients failed to improve within two weeks and/or required emergent surgical intervention at any point, even beyond the initial two weeks. Additionally, if corneal cultures were collected, the rate of culture positivity, specific organism cultured, and sensitivity to a standard panel of antimicrobial agents as reported by the clinical microbiology laboratory were documented. Of note, the corneal culture technique was not specifically documented in the medical record, but standard practice at the University of Rochester included either Kimura platinum spatula (Storz) or Eswab (Copan), the latter of which was introduced in 2019. While standard practice suggests obtaining cultures in cases of large ulcers (>5mm) or in refractory cases, ultimately the decision to obtain cultures was based on individual clinician’s judgement. Clinical microbiology results were generated from standard, growth-based microbiology culture techniques. In culture-negative cases, the diagnosis of IK was determined based on clinical judgment of the provider.

Slit lamp exam findings were utilized to determine whether an ulcer is “sight threatening” (high risk for causing loss of visual acuity of 2 or more Snellen lines) (34). Probably Sight Threatening (PST) ulcers were categorized according to the “’1,2,3 rule,” as those with one or more of the following high-risk characteristics: >/=1+ cell in the anterior chamber, ulcer is >/=2mm in greatest linear dimension and/or edge of the infiltrate is </=3 mm from the center of the cornea. In contrast, Rarely Sight Threatening (RST) ulcers meet none of the above criteria (34).

### Statistical analysis

Descriptive statistics were computed to summarize patients’ demographics and clinical characteristics. For each outcome, multivariable logistic regression was used to assess its association with a set of pre-selected risk factors, which is slightly different across different outcome variables. An odds ratio of each regression coefficient and the corresponding 95% confidence interval were computed. Given the exploratory nature of this study, multiple testing was not corrected. For all analyses, a significance level of 0.05 (two-sided) was used to consider statistical significance. All data analyses were performed using R (version 4.1.3) (35).

## Results

From April 2012 to May 2022, a total of 1651 patients were initially identified based on ICD diagnostic codes relating to infectious keratitis. Of these, 935 patients were verified as having true infectious keratitis at the University of Rochester Medical Center (**Figure 1**).

### Patient characteristics

The sociodemographic, medical history and ocular history of this population is summarized in **Table 1**. The mean age of patients was 48.6 ± 20.8 years old with 262 (27.9%) patients younger than 35, 448 (47.8%) patients between 35-65, and 225 (24%) patients older than 65. Females were slightly more prevalent (n=492, 52.5%) than males (n=443, 47.3%). Racial analysis was based on self-identification and included 729 (77.9%) Caucasian patients, 110 (11.7%) patients who identified as African American or Black, 23 (2.4%) Asian, and 33 (3.5%) patients that did not wish to self-identify. Additionally, 41 (4.3%) patients identified as “other” which included diverse groups such as Alaska native, Hawaiian native and Middle Eastern. The ethnicity of patients was predominantly Non-Hispanic or Latino (n=779, 83.2%). The primary language of patients was English (n=898, 95.9%), with fewer non-English speakers (n=37, 3.9%) identified as patients requiring language interpretation for the clinical visit.

**Table 1:** Demographics, medical and ocular history of patients included in this study.

Characteristic	n (%)
Total	935 (100%)
*Sociodemographic Variables*	
Age	
<35	262 (27.9%)
35-65	448 (47.8%)
>65	225 (24%)
Sex	
Male	443 (47.3%)
Female	492 (52.5%)
Race	
Caucasian	728 (77.9%)
Black	110 (11.7%)
Asian	23 (2.4%)
Other	41 (4.3%)
Not specified	33 (3.5%)
Ethnicity	
Not Hispanic or Latino	779 (83.2%)
Hispanic or Latino	45 (4.8%)
Not specified	111 (11.9%)
Language	
English	898 (95.9%)
Not English	37 (3.9%)
Insurance Coverage	
Commercial	524 (55.9%)
Medicare	235 (25.1%)
Medicaid	107 (11.4%)
Self-pay	69 (7.3%)
Distance to URMC	
<15 miles	433 (46.3%)
15-60 miles	286 (30.4%)
>60 miles	216 (23.1%)
*Medical History*	
Hypertension	302 (32.2%)
Diabetes	151 (16.1%)
Diabetes Control	
Good	58 (38.4%)
Poor	66 (43.7%)
Unknown	24 (15.9%)
Smoker	401 (42.8%)
Systemic Immunosuppressive Medications	92 (9.8%)
*Ocular History*	
Contact Lens Use	482 (51.4%)
Ocular Surface Disease	84 (9.0%)
History of Ocular Trauma	166 (17.7%)
Prior Intraocular Surgery (any)	235 (25.1%)
Corneal Transplant	74 (7.9%)
Glaucoma	121 (12.9%)
Prior Infectious Keratitis	125 (13.3%)
BCVA at Baseline	
Mild or No Visual Impairment	405 (43.2%)
Moderate Visual Impairment	52 (5.5%)
Severe visual Impairment	67 (7.1%)
No known baseline VA	411 (43.9%)

More than half of patients had commercial insurance (n=524, 55.9%), followed by Medicare (n=235, 25.1%), then Medicaid (n=107, 11.4%) and self-pay (n=69, 7.3%). Lastly the distance from home zip code to URMC was calculated, to explore the catchment area of patients and consider the potential role of transportation as a barrier to care. A close distance to URMC was defined as within 15 miles (n=433, 46.3%), medium distance within 15 – 60 miles (n=286, 30.4%), and far distance >60 miles (n=216, 23.1%).

With regards to medical history, approximately a third of patients had reported diagnoses of hypertension (n=302, 32.2%), while 16.1% had a diagnosis of diabetes (n=151). Of those patients, 58 (38.4%) had good control of their diabetes, defined as a HbA1c<7% within the past year, while 66 (43.7%) had poor control, and 24 (15.9%) were unknown. 42.8% of patients (n=401) were current or previous smokers, with at least a 5 pack-year history. Patients’ medication list was examined for any current use of immunosuppressant medications and 92 patients (9.8%) were found to be taking such medications.

With regards to ocular history, 51.4% of patients were contact lens users within the two weeks prior to presentation (n=482). 9% of patients (n=84) were classified with prior or concurrent ocular surface disease. Ocular trauma, defined as a history of traumatic injury at any point prior to presentation, was reported in 166 patients (17.7%). 235 patients (25.1%) had previous intraocular surgery, including 74 patients (7.9%) who had prior history of corneal transplant. 121 patients (12.9%) had prior diagnoses of glaucoma, with 80 of those patients (66.1%) on topical medications at the time of presentation for IK. Meanwhile, 125 patients (13.3%) had a prior history of infectious keratitis, which was defined as any bacterial, fungal or viral corneal infection that was fully resolved prior to presentation. A baseline BCVA was recorded for any patients seen within 1 year prior to presentation. There were 411 patients (43.9%) with no known baseline VA. Otherwise, patients’ baseline BCVA was categorized according to the World Health Organization categories of visual impairment: 405 patients (43.2%) with mild or no visual impairment (≥20/70 with correction), 52 patients (5.5%) with moderate visual impairment (Between 20/70 and 20/200) and 67 patients (7.1%) with severe visual impairment or blindness (< 20/200).

### Clinical presentation and treatment course

**Table 2** summarizes patients’ clinical presentation and treatment course. Most patients (391, 41.8%) presented with mild or no visual impairment, 198 (21.2%) presented with moderate visual impairment, while 346 (37%) presented with severe visual impairment. 858 patient records included measurements of the ulcer size. Of those, small ulcers (<2 mm in widest diameter) were reported in 448 patients (47.8%), while 312 patients (33.3%) had medium ulcers (2-5 mm) and 98 (10.4%) had large ulcers (>5 mm). Ulcer severity was determined on the “1,2,3 Rule”^33^ and 893 patients had sufficient data to be evaluated by this rule. Based on these parameters, 534 patients (59.8%) had Probably Sight Threatening (PST) ulcers, 359 patients (40.2%) had Rarely Sight Threatening (RST) ulcers. Across all patients, 485 (51.9%) had microbiology cultures collected at presentation, and of those cultured, 320 resulted in positive growth. Among positive cultures, 41.9% grew Gram-positive bacteria (n=134), of which the most common organisms were *Staphylococcus aureus* (n=43), and coagulase-negative *Staphylococcus spp* (n=95), 20.3% Gram-negative bacteria (n=65), of which the most common organisms were *Pseudomonas aeruginosa* (n=48), and *Serratia* spp. (n=10), 4.7% fungal (n=15), 2.5% viral (n=8) and 2.2% protozoa (n=7). Importantly, 30.6% of positive cultures resulted in polymicrobial growth (n=98). 178 cultured organisms displayed antibiotic resistance to one or more antimicrobial class (55.5% of all positive cultures). Of these, 96 were considered multidrug resistant, with resistance to three or more antimicrobial classes (30% of positive cultures).

**Table 2:** Clinical characteristics of IK patients on presentation.

Characteristic	n (%)
BCVA at Presentation	
Mild or No Visual Impairment	391 (41.8%)
Moderate Visual Impairment	198 (21.2%)
Severe visual Impairment	346 (37.0%)
Ulcer Size	
<2 mm	448 (47.8%)
2-5 mm	312 (33.3%)
>5 mm	98 (10.4%)
Ulcer Severity	
PST	534 (59.8%)
RST	359 (40.2%)
*Clinical Course*	
Cultures Collected	485 (51.9%)
Cultures with Growth	320 (65.9%)
Cultured Organism Class	
Gram Positive	134 (41.9%)
Gram Negative	65 (20.3%)
Fungus	15 (4.7%)
Virus	8 (2.5%)
Protozoa	7 (2.2%)
Polymicrobial	98 (30.6%)
Antibiotic Resistance to >1 Antibiotic Class	178 (55.5%)
Antibiotic Resistance to >3 Antibiotic Classes	96 (30%)
Initial Treatment with Fluoroquinolone	458 (48.9%)
Initial Treatment with Compounded Antibiotics^‡^	361 (38.5%)
>2 Weeks to Heal	108 (11.6%)
Surgical Intervention	99 (10.5%)
Treatment Failure	145 (15.5%)
Lost to Follow-Up	195 (20.9%)

^‡^Compounded antibiotics included vancomycin 25mg/ml and tobramycin 14mg/ml.

To further describe the antibiotic resistance profile of those organisms that resulted from positive cultures, the antibiogram for the two most common causes of Gram-positive and -negative organisms is displayed in **Table 3**. *S. aureus* displayed high amounts of resistance to cefazolin (28% resistant), erythromycin (54%), levofloxacin (28%), moxifloxacin (26%), and methicillin (26%). A similar pattern was noted among coagulase-negative *Staphylococci*, with significant resistance noted against cefazolin (36%), erythromycin (52%), levofloxacin (11%), moxifloxacin (13%), methicillin (35%), and tetracycline (14%). Of note, resistance among Gram-negative organisms was low across all antibiotics tested, except for cefazolin towards *Serratia*, which would be expected given the spectrum of activity of this drug. Moreover, multidrug resistance was highly concordant with methicillin resistance, as 92% of all methicillin-resistant strains were also found to be multidrug resistant.

**Table 3:** Percent resistance rates among the most commonly identified organisms.

Drug	*S. aureus*	Coagulase negative *Staph.*	*P. aeruginosa*	*Serratia* spp.
Cefazolin	28	36	2	40
Doxycycline	0	1	0	0
Erythromycin	54	52	6	0
Gentamicin	0	1	0	0
Levofloxacin	28	11	2	0
Moxifloxacin	26	13	2	0
Ciprofloxacin	0	0	6	0
Methicillin	26	35	2	0
Tetracycline	5	14	0	0
Tobramycin	0	1	0	0
Vancomycin	0	0	0	0

The most common initial therapeutic regimens included fluoroquinolone monotherapy (such as moxifloxacin 0.5%), prescribed to 458 patients (48.9%) and combination therapy with compounded vancomycin 25 mg/ml and tobramycin 14mg/ml (n=361, 38.5%). Other utilized therapeutic regimens included commercially available tobramycin combined with dexamethasone and polymyxin B 10 000u/ trimethoprim 1mg/ml combination drops.

To assess response to treatment, the time to clinical improvement—defined as clinician documentation of clinical improvement or decreased size of ulcer on slit lamp examination—was recorded. 108 patients (11.6%) had slow resolution of symptoms, requiring two weeks or longer to demonstrate signs of clinical improvement. Furthermore, 99 patients (10.5%) required surgical or procedural intervention (including corneal transplant, corneal gluing, or a corneal patch graft). As such, for the purposes of this study, treatment failure was defined as any patient who had slow resolution of symptoms (>2 weeks for clinical improvement) or who required surgical intervention. In total, 145 patients (15.5%) met this definition of treatment failure. Of the cases of treatment failure, 6.90% (n=10) were fungal infections, 2.76% (n=4) were acanthamoeba, 2.07% (n=3) were viral and the remaining 88.27% (n=128) had a bacterial etiology. A total of 195 patients (20.9%) were deemed lost to follow up if there were no final visits documenting resolution of IK.

### Treatment failure in infectious keratitis

To understand the impact of individual factors (sociodemographic and clinical) on treatment failure as well as control for confounding variables, a logistic regression analysis was performed using clinically relevant variables. As shown in **Table 4**, older age was found to have increased risk for treatment failure (OR 1.68, p=0.006), while Medicare insurance (compared to Medicaid) was protective (OR 0.37, p=0.05). Of note, although the calculated odds ratio for treatment failure was >1 for race (Black vs White, Asian vs White, Other vs White) this did not reach the level of statistical significance. Hispanic ethnicity compared to non-Hispanic ethnicity had a calculated odds ratio <1, but non-significant p-value. Similarly, in this logistic regression model, the calculated odds ratio was >1 for increased geographic distance from URMC but was not found to be statistically significant.

**Table 4:** Treatment Failure, Multivariate Logistic Regression Analysis.

Characteristic	Odds Ratio	95% CI	P-value
*Sociodemographic Variables*			
Age	1.68	1.17 – 2.46	0.006*
Male Sex	1.25	0.74 – 2.12	0.408
Race, vs White			
Black	1.50	0.62 – 3.46	0.354
Asian	2.89	0.37 – 14.78	0.244
Other	2.43	0,69 – 8.40	0.159
Hispanic Ethnicity	0.26	0.05 – 1.16	0.093
Insurance Status, vs Medicaid			
Commercial	0.41	0.17 – 1.04	0.051
Medicare	0.37	0.14 – 1.00	0.050*
Self-Pay	0.31	0.07 – 1.14	0.088
Distance from URMC, vs Short (<15 mi)			
Medium distance (15-60 mi)	1.34	0.69 – 2.62	0.387
Long distance (>60 mi)	1.92	0.98 – 3.78	0.056
*Medical History*			
Good Diabetic Control (HbA1c<7)	1.40	0.59 – 3.68	0.472
Smoker	0.97	0.58 – 1.62	0.895
Systemic Immunosuppressive Medications	1.94	0.93 – 3.96	0.071
*Ocular History*			
Contact Lens Use	0.48	0.26 – 0.88	0.019*
Prior Intraocular surgery (any)	1.47	0.75 – 2.88	0.259
Corneal Transplant	2.58	1.18 – 5.70	0.018*
Glaucoma	0.86	0.43 – 1.70	0.662
BCVA at Presentation, vs mild or no visual impairment			
Moderate visual impairment	2.19	0.91 – 5.49	0.085
Severe visual impairment	4.72	2.13 – 11.20	<0.001*
Ulcer Size, vs small (<2 mm)			
Medium (2-5 mm)	1.56	0.81 – 3.04	0.186
Large (>5 mm)	3.36	1.50 – 7.61	0.003*
Antibiotic Resistance to >3 Antibiotic Classes	1.23	0.60 – 2.46	0.562
Organism Class, vs Bacterium			
Fungus	7.37	2.20 – 25.44	0.001*
Protozoan	15.54	1.88 – 125.74	0.009*
Virus	0.49	0.06 – 2.78	0.445

*Statistically significant values.

Medical history factors such as diabetic control, smoking status and use of systemic immunosuppressive medications were also not found to be statistically associated with treatment failure when controlling for confounding variables, although immunosuppression did show a trend towards higher risk of treatment failure (OR 1.94, p=0.07). In contrast, there were several ocular history variables that were associated with a higher risk of treatment failure including a history of a corneal transplant (OR 2.58, p=0.018), severe visual impairment on presentation (OR 4.72, p<0.001) and large size of ulcer on presentation (OR 3.36, p=0.003). Treatment failure in patients presenting with moderate visual impairment approached significance (OR 2.19, p=0.085). Of note, contact lens wearers were less likely to experience treatment failure (OR 0.48, p=0.019).

With regards to the causative organism, there was a higher risk of treatment failure in both fungal (OR 7.37, p=0.001) and protozoan (OR 15.54, p=0.009) cases compared to bacterial causes. Among bacterial causes, multidrug resistance was not found to be statistically associated with treatment failure.

### Clinical decision making in infectious keratitis

In addition to understanding IK treatment failure in the context of patient sociodemographic and clinical history/presentation characteristics, we next sought to further understand how these variables may also impact clinical decision making. In this regard, additional logistic regression analyses were performed to explore the decision to collect microbiology cultures and pursue surgical intervention. As shown in **Table 5**, patients who identified as Black were less likely to have corneal cultures compared to White patients (OR 0.52, p=0.037) whereas patients who identified as Other Race were more likely to receive cultures than White patients (OR 4.80, p=0.005). Additionally, patients with moderate visual impairment (OR 1.81, p=0.0133), severe visual impairment (OR 5.04 p<0.001), medium-sized ulcers (OR 6.25, p=<0.001), and large ulcers (OR 7.10, p<0.001) were more likely to receive cultures.

**Table 5:** Cultures Collected, Multivariate Logistic Regression Analysis.

Characteristic	Odds Ratio	95% CI	P-value
*Sociodemographic Variables*			
Age	1.07	0.82 – 1.40	0.610
Male Sex	1.26	0.85 – 1.86	0.246
Race, vs White			
Black	0.52	0.28 – 0.96	0.037*
Asian	0.53	0.14 – 2.03	0.355
Other	4.80	1.59 – 14.48	0.005*
Hispanic Ethnicity	0.71	0.25 – 2.04	0.527
Insurance Status, vs Medicaid			
Commercial	0.98	0.51 – 1.88	0.953
Medicare	0.75	0.33 – 1.67	0.479
Self-Pay	1.11	0.45 – 2.75	0.828
Distance from URMC, vs short (<15 mi)			
Medium distance (15-60 mi)	0.88	0.56 – 1.39	0.591
Long distance (>60 mi)	0.81	0.48 – 1.37	0.429
*Medical History*			
Good Diabetic Control (HbA1c<7)	0.53	0.25 – 1.12	0.096
Smoker	1.23	0.83 – 1.82	0.302
Systemic immunosuppressant medications	1.07	0.55 – 2.09	0.835
*Ocular History*			
Contact Lens Use	1.43	0.91 – 2.24	0.124
Prior Intraocular Surgery (any)	0.87	0.48 – 1.56	0.634
Corneal Transplant	1.37	0.55 – 3.39	0.494
Glaucoma	1.37	0.70 – 2.70	0.357
BCVA at presentation, vs mild or no visual impairment			
Moderate visual impairment	1.81	1.13 – 2.90	0.0133*
Severe visual impairment	5.04	3.02 – 8.41	<0.001*
Ulcer Size, vs small (<2 mm)			
Medium (2-5 mm)	6.25	4.09 – 9.55	<0.001*
Large (>5 mm)	7.10	3.28 – 15.34	<0.001*

*Statistically significant values.

Another critical clinical decision point in the management of infectious keratitis is whether to pursue surgical intervention. As shown in **Table 6**, among the sociodemographic variables tested, patients who identify as Other Race were more likely to receive surgery (OR 4.66, p=0.023) than White patients with similar characteristics. Additionally, patients who lived greater than 60 miles from URMC were more likely to receive surgery than those who lived closer (OR 2.10, p=0.051). Medical history variables such as diabetes, smoking status and use of systemic immunosuppressive medications were not statistically associated with surgical intervention. However, with regards to ocular history or clinical presentation, patients who had had a prior corneal transplant were more likely to receive surgical intervention (OR 2.48, p=0.027), as were patients presenting with moderate (OR 11.70, p<0.001) or severe visual impairment (OR 22.21, p<0.001). Similarly, patients presenting with medium-sized ulcers (OR 2.22, p=0.044) or large ulcers (OR 5.79, p<0.001) had a greater likelihood of receiving surgery than those with small ulcers. Interestingly, patients with a history of contact lens use were less likely to receive surgery (OR 0.32, p<0.001) compared to those patients without this pertinent history. While fungal and protozoan infections had higher risk of treatment failure, this was a result of prolonged medical treatment (>2 weeks) rather than surgical intervention. Indeed, neither the class or organisms or multidrug resistant status of bacteria were significantly correlated with surgical intervention.

**Table 6:** Surgical Intervention, Multivariate Logistic Regression Analysis.

Characteristic	Odds Ratio	95% CI	P-value
*Sociodemographic Variables*			
Age	1.21	0.81 – 1.83	0.349
Male Sex	1.17	0.65 – 2.12	0.594
Race, vs White			
Black	1.85	0.69 – 4.82	0.218
Asian	2.60	0.23 – 16.26	0.383
Other	4.66	1.24 – 18.66	0.023*
Hispanic Ethnicity	0.23	0.03 – 1.17	0.079
Insurance Status, vs Medicaid			
Commercial	0.84	0.28 – 2.82	0.758
Medicare	0.78	0.24 – 2.76	0.683
Self-Pay	0.99	0.23 – 4.29	0.991
Distance from URMC, vs short (<15 mi)			
Medium distance (15-60 mi)	1.51	0.71 – 3.26	0.289
Long distance (>60 mi)	2.10	1.00 – 4.48	0.051
*Medical History*			
Good Diabetic Control (HbA1c<7)	2.22	0.82 – 7.16	0.121
Smoker	1.07	0.60 – 1.90	0.820
Systemic Immunosuppressive Medications	2.12	0.95 – 4.59	0.065
*Ocular History*			
Contact Lens Use	0.32	0.15 – 0.64	0.001*
Prior Intraocular Surgery (any)	1.49	0.69 – 3.21	0.306
Corneal Transplant	2.48	1.11 – 5.64	0.027*
Glaucoma	0.87	0.41 – 1.80	0.712
BCVA at presentation, vs mild or no visual impairment			
Moderate visual impairment	11.70	2.60 – 111.54	<0.001*
Severe visual impairment	22.21	5.41 – 204.27	<0.001*
Ulcer Size, compared to small (<2 mm)			
Medium (2-5 mm)	2.22	1.02 – 5.21	0.044*
Large (>5 mm)	5.79	2.37 – 15.08	<0.001*
Antibiotic Resistance to >3 Antibiotic Classes	1.23	0.57 – 2.55	0.586
Organism Class, vs Bacterium			
Fungus	2.62	0.69 – 9.14	0.151
Protozoan	0.46	0.00 – 7.62	0.632
Virus	0.36	0.03 – 2.09	0.275

*Statistically significant values.

A final logistic regression was performed to understand if any patient characteristics were associated with a presentation of a Rarely Sight Threatening (RST) vs. a Probably Sight Threatening (PST) ulcer, after controlling for confounders (**Table 7**). In this analysis, older age predicted a lower likelihood of presenting with a RST ulcer, or in other words more likely to present with more severe disease (OR 0.751, p=0.0089). Additionally, those patients who lived a long-distance from URMC were also more likely than those who are a short distance away (OR 0.459, p<0.001) to present with more severe disease. Of note, there was a trend of presenting with a PST ulcer among patients with Medicare (OR 0.560, p=0.0698) and a PST ulcer among those who were classified as self-pay (OR 0.481, p=0.0545). Among medical and ocular variables, only those patients with a history of intraocular surgery were more likely to present with a more severe ulcer (OR 0.504, p=0.006) than those without this surgical history.

**Table 7:** Ulcer Severity, Multivariate Logistic Regression Analysis. Lower odds ratio predictive of RST ulcer.

Characteristic	Odds Ratio	95% CI	P-value
*Sociodemographic Variables*			
Age	0.75	0.61 – 0.93	0.009*
Male Sex	0.93	0.68 – 1.27	0.642
Race, vs White			
Black	0.76	0.47 – 1.24	0.273
Asian	0.92	0.33 – 2.58	0.877
Other	0.82	0.35 – 1.93	0.651
Hispanic Ethnicity	1.44	0.64 – 3.24	0.382
Insurance Status, vs Medicaid			
Commercial	0.73	0.44 – 1.22	0.228
Medicare	0.56	0.30 – 1.05	0.070
Self-Pay	0.48	0.22 – 1.01	0.055
Distance from URMC, vs short (<15 mi)			
Medium distance (15-60 mi)	0.81	0.56 – 1.16	0.250
Long distance (>60 mi)	0.46	0.30 – 0.71	<0.001*
*Medical History*			
Good Diabetic Control (HbA1c<7)	1.38	0.73 – 2.60	0.321
Smoker	1.06	0.77 – 1.47	0.711
Systemic Immunosuppressive Medications	1.49	0.87 – 2.54	0.145
*Ocular History*			
Contact Lens Use	0.99	0.70 – 1.40	0.942
Prior Intraocular Surgery (any)	0.50	0.31 – 0.82	<0.001*
Corneal Transplant	0.74	0.34 – 1.62	0.454
Glaucoma	0.76	0.43 – 1.33	0.336

*Statistically significant values.

## Discussion

Among the 935 IK cases included in this study, the majority of patients were between the ages of 35-65, Caucasian, non-Hispanic, English-speaking, held commercial insurance and lived within 15 miles of the URMC. There were roughly equal numbers of males and female patients. Overall, approximately one third of patients had hypertension, 16.1% had diabetes, and 9.8% were on systemic immunosuppression. Slightly less than half were smokers. Of note, the rates of hypertension, diabetes, and smoking among patients in this study approximately mirror the broader population of Monroe County (36–38), in which URMC and the city of Rochester is located. Similarly, 7.38% of patients in this study were uninsured and 37.21% had public health insurance (Medicaid, Medicare or VA), which closely aligns with the city of Rochester, in which 7.4% are uninsured and 38% receive at least some public health insurance coverage (39). In contrast, other demographic rates vary significantly. Specifically, while 78% of patients in our study were Caucasian, the 2023 United States Census estimated that Rochester is comprised of a more diverse racial and ethnic makeup that is 43.2% White people, 37.9% Black people, and 19.7% Hispanic people. This discrepancy between the patients in this study compared to surrounding community is likely multifactorial and could include perceived and true barriers to accessing care at an academic medical center related to transportation, insurance, implicit and explicit bias, and broader distrust in the medical system (40, 41).

With regards to prior ocular history, 51.4% of patients in this study were current contact lens wearers, which is consistent with the well-established risk of IK in this population of patients (18, 20, 21). Perhaps reflecting the tertiary ophthalmology practice at URMC, 7.9% of this cohort had a prior corneal transplant, 13% had concurrent glaucoma, and 13.3% had a prior diagnosis of IK. Corneal transplant status is also an established risk factor for IK, with previously reported rates of microbial keratitis post-transplant ranging from 1.76% to 7.4% (42–45), likely secondary to chronic topical corticosteroid use, suture related infections, or perturbations of the ocular surface. Not much is known about the risk of IK among existing glaucoma patients, however it is possible that chronic use of topical medications and subsequent ocular surface disease may increase the risk of developing IK. Similarly, it is not exactly known why some patients who have had IK previously may be more likely to develop IK in the future, but this could be related to underlying patient characteristics such as contact lens wear, ocular surface disease or immunosuppression.

Overall, there was a wide range of severity of IK cases that presented over the course of this study. While only 10.4% of patients presented with an ulcer greater than 5mm, 57% of patients were categorized as having a probably sight threatening ulcer as defined by the “1,2,3 rule” (34). Interestingly, only approximately half of the patients had cultures collected at the initial visit. Of these, 66% exhibited positive growth, which is similar to culture positivity rates reported in other studies at other academic centers (46, 47), and 62% of cultures were bacterial. Among bacterial cases, our study found high antibiotic resistance rates with 55.5% of cultured bacteria displaying resistance to at least one antibiotic and 30% displaying multidrug resistance. Interestingly, while Gram-negative organisms were generally found to be sensitive to commonly used ophthalmic antibiotics, significant rates of resistance to erythromycin, cefazolin and fluoroquinolones were found among Gram-positive bacteria. Well-aligned with the American Academy of Ophthalmology guidelines on the treatment and management of bacterial keratitis (48), the most common empiric treatment of IK at URMC was a fluoroquinolone antibiotic, while the next most common regimen was a combination of compounded vancomycin 25mg/ml and tobramycin 14mg/ml, both of which resulted in a high treatment success rate in our patient population. However, the high rates of antibiotic resistance among ocular bacterial strains observed at URMC as well as globally, especially in developing countries, raises concern over our continued ability to eradicate infections with our current armamentarium (49–51). While fluoroquinolones are favored due to their broad-spectrum activity, availability, and patient tolerability, their widespread use has made this class of drugs particularly vulnerable. Compounded vancomycin and tobramycin remain good alternatives but are not widely available due to the lack of commercial formulations. As such there is a critical need for new ophthalmic antimicrobial therapeutics.

This study also sought to identify clinical and sociodemographic risk factors that portend poor outcomes for patients as defined by greater than 2 weeks to heal and/or the need for surgical intervention. In the presented multivariate analysis, after controlling for confounding variables, age, prior corneal transplant, presenting vision <20/200, a large ulcer (presenting ulcer size of >5mm), and infection with a fungal or protozoan organism were noted to be significantly associated with an increased likelihood treatment failure. In general, our findings are consistent with other studies. For example, older age has been well established as a risk factor for poor outcomes in IK (9, 23, 52–54), and large ulcer size (>5mm) has also been shown to increase the risk of corneal perforation, surgical intervention and worsening clinical signs across several international studies (9, 53–55). Similarly, significant vision impairment at the time of presentation has been previously linked to subsequent treatment failure in IK (55–57). With respect to the infecting organisms, it is well accepted that fungal and protozoan infections are notoriously difficulty to treat and thus it is unsurprising that these patients had a higher risk of treatment failure compared to bacterial keratitis in this study (54, 58, 59).

In efforts to further understand those variables that may impact clinical decision making in IK, we investigated the variables associated with the likelihood of obtaining microbiologic cultures and receiving surgical intervention. With regards to cultures, unsurprisingly, patients presenting with increasingly severe vision and/or large ulcers were more likely to have microbiology cultures collected at the first visit as well as subsequent surgical interventions. However, importantly, after considering all clinical variables, Black patients were less likely to receive cultures than White patients. How race or other demographic factors (for example, age, sex, ethnicity) may impact clinical decision making in ophthalmic care, particularly in diabetic retinopathy and glaucoma, is becoming increasingly recognized (60–62). With respect to the clinical management of IK, race and other social determinants of health are just now being reported. For example, in a recently published study, non-White patients had fewer visits and procedures during their treatment course for IK compared to White patients (33).

Finally, we sought to understand if certain patient characteristics were associated with a higher probability of presenting with a PST (probably sight threatening) ulcer. In this analysis, older age and a home address greater than 60 miles from URMC were statistically associated with PST ulcers. This may reflect a variety of social determinants of health including transportation, lack of independent living, cost, or other co-morbidities. Patients with ocular comorbidities were more likely to present with PST ulcers. For instance, 3 out of every 4 patients with a history of prior intraocular surgery presented with PST ulcers. This could be a direct reflection of ocular comorbidities as well as the increased risk associated with certain medications, such as topical steroids following a prior corneal transplant.

While this study is the first in Western New York to comprehensively describe IK, there are several limitations. First, based on the inconsistent use and results of microbial cultures, 615 cases in this study were based on clinical diagnosis (i.e. not proven); thus, it is possible that there are patients included in this analysis that were not true cases of IK. Additionally, some patient entries were incomplete due to the retrospective nature of this study thereby potentially impacting the strength of the conclusions. Lastly, a pre-selected list of risk factors was used in the presented multivariate regressions thereby introducing some bias into the analysis. Ultimately, though, it will be critical for the field to continue expanding our understanding of regional differences in risk factors, clinical outcomes and management of IK. A deeper appreciation of sociodemographic variables and social determinants of health will provide a more holistic lens so that true advances in both the diagnosis and management of IK can be achieved.

## Data Availability

The original contributions presented in the study are included in the article/**Supplementary Material**. Further inquiries can be directed to the corresponding author/s.
