# Supplementary material for: Infectious keratitis in Western New York: a 10-year review of patient demographics, clinical management, and treatment failure
Source: Front Ophthalmol (Lausanne). 2024 Dec 11;4:1469966. doi: 10.3389/fopht.2024.1469966 (PMC11668731; doi:10.3389/fopht.2024.1469966)
Supplement: Supplementary file 1 [file Table1.docx]

| **Supplemental Table 1. ICD 9 and 10 codes with corresponding diagnoses, which were used in patient selection.** | | |
| --- | --- | --- |
| **Primary Diagnosis (ICD 10)** | **ICD-10** | **ICD-9** |
| **Corneal ulcer** | H16.009 | 370 |
| **Central corneal ulcer** | H16.019 | 370.03 |
| **Marginal corneal ulcer** | H16.049 | 370.01 |
| **Mycotic corneal ulcer** | H16.069 |  |
| **Keratitis** | H16.9 | 370.9 |
|  |  | 370.8 |
| **Keratitis due to fungus** | H16.8 |  |
|  | B49 |  |
| **Keratitis due to infection** | H16.9 |  |
| **Keratitis due to microsporidia** | H16.8 |  |
|  | B48.8 |  |
| **Keratitis due to syphilis** | A50.31 | 090.3 |
| **Keratitis due to trauma** | H16.9 |  |
|  | H16.001 |  |
| **Keratitis of both eyes due to Acanthamoeba species** | B60.13 | 136.21 |
|  | H16.003 |  |
|  | H16.002 |  |
|  | A31.9 |  |
| **Acanthamoeba infection: disseminated/endophthalmitis** | B60.10 |  |
|  | B60.19 |  |
|  | H44.19 |  |
| **Keratitis of both/left/right eyes due to adenovirus** | B30.0 |  |
| **Keratitis of both/left/right eyes due to bacteria** | H16.8 |  |
|  | B96.89 |  |
| **Keratitis of both/left/right eyes due to VZV** | B02.33 |  |
| **Keratitis 2/2 contact lens use** | H16.8 | 077.1 |
|  | H18.829 |  |
|  | H18.219 |  |
|  | H18.821 |  |
|  | H18.213 |  |
| **Disorder of cornea 2/2 CL use** | H57.89 | 371.82 |
|  | H18.823 | 371.24 |
|  | H18.822 |  |
| **Keratitis, dendritic** | H16.329 |  |
|  | H16.321 |  |
| **Keratitis, diffuse interstitial** | H16.329 | 370.52 |
|  | H16.323 |  |
|  | H16.322 |  |
| **Corneal graft infection** | T86.8429 |  |
| **Ulcer of cornea 2/2 bacterial infection** | B96.89 |  |
|  | T86.8429 |  |
| **Corneal transplant infection** | T86.8423 | 918.1 |
|  | T86.8422 | 918.2 |
|  | T86.8421 |  |
|  | S05.02XD |  |
|  | S05.01XA |  |
|  | S05.01XS |  |
|  | S05.01XD |  |
|  | S05.00XS |  |
|  | S05.00XD |  |
| **Abrasion of cornea with infection** | S05.00XA | 371.89 |
|  | S05.02XA |  |
|  | S05.02XS |  |
| **Fungal infection of cornea** | B49 |  |
|  | H18.899 |  |
|  | H57.89 |  |
| **HSV post-op infection of cornea** | H59.89 | 054.40 |
|  | B00.50 |  |
| **HSV keratitis** | B00.52 | 054.42 |
|  |  | 054.43 |
|  |  | 053.21 |
| **Infection of cornea** | B99.9 |  |
|  | H44.009 |  |
| **Eye infection** | H44.003 |  |
|  | H44.002 |  |
|  | H44.001 |  |
| **Gonococcal eye infection** | H05.10 | 098.49 |
|  | A54.39 |  |
|  | A54.30 |  |
|  | H44.129 |  |
| **Parasitic eye infection** | H44.123 |  |
|  | H44.122 |  |
|  | H44.121 |  |
|  | H44.001 |  |
|  | B97.89 |  |
| **Viral infection of eye** | H44.129 |  |
|  | H44.003 |  |
|  | H44.002 |  |
| **Disorder of eyeball with HIV infection** | B20 |  |
|  | H44.9 |  |
| **HSV simplex ophthalmicus** | B00.50 |  |
| **Herpes zoster ophthalmicus** | B02.30 | 053.29 |
| **Infection of eye 2/2 avian paramyxovirus 1** | B97.89 |  |
| **Infection of eye 2/2 borrelia** | A69.8 |  |
| **Infection of eye 2/2 MRSA** | A49.02 |  |
| **Corneal scar** | H17.9 |  |
